# Prevalence and Genotyping of *Trichomonas vaginalis* Infected to dsRNA Virus by PCR–Restriction Fragment Length Polymorphism (RFLP)

**Published:** 2019

**Authors:** Fariba ORUJZADEH, Fatemeh TABATABAIE, Khadijeh KHANALIHA, Lame AKHLAGHI, Farah BOKHARAEI-SALIM, Soudabeh FALLAH, Abdoulreza ESTEGHAMATI, Hossein MASOUMI-ASL

**Affiliations:** 1. Department of Parasitology and Mycology, School of Medicine, Iran University of Medical Sciences, Tehran, Iran; 2. Research Center of Pediatric Infectious Diseases, Institute of Immunology and Infectious Diseases, Iran University of Medical Sciences, Tehran, Iran; 3. Department of Virology, School of Medicine, Iran University of Medical Sciences, Tehran, Iran; 4. Department of Clinical Biochemistry, School of Medicine, Iran University of Medical Sciences, Tehran, Iran

**Keywords:** Genotype, Actin, RFLP, *Trichomonas vaginalis*

## Abstract

**Background:**

*Trichomonas vaginalis* is a prevalent sexually transmitted infection cause trichomoniasis. In this study prevalence and genotype of Iranian isolates of *T. vaginalis* infected (dsRNA) viruses were evaluated by PCR-RFLP and obtained patterns were then confirmed by sequence analysis and genotype of these Iranian isolates confirmed again.

**Methods:**

Ten strains of *T.vaginalis* were collected from 1700 vaginal samples of women referred to hospitals associated with Iran University of Medical Sciences in Tehran, Iran during Feb 2016 to Jul 2017, evaluated in points of infection to *T. vaginalis* Virus (TVV-1) were used in a PCR-RFLP. All of ten isolates of T. vaginalis were examined by designed nested PCR for actin gene and then digestion patterns of three endonuclease enzymes of HindII, MseI and RsaI were evaluated and genotype of these isolates was defined.

**Results:**

By combination of fragments pattern of three enzymes of HindII, RsaI and MseI, three genotypes were found; six genotypes E, two genotypes G and two genotypes I. The most dominant genotypes were genotype E. Among four TVV infected isolates two genotype E, one genotype G and one genotype I were found, however among six uninfected *T. vaginalis* isolates to TVV-1, all of three genotypes were also found.

**Conclusion:**

Three genotypes E, G and I in *T. vaginalis* infected with dsRNA isolates were found, however, these three genotypes in *T. vaginalis* without virus were also observed. Further study is needed to evaluate genotypes of *T. vaginalis*, which infected virus in more great T. vaginalis population.

## Introduction

*Trichomonas vaginalis* is urogenital protozoan that is the cause of trichomoniasis. The common manifestations are discharge, irritation, dysuria, flagrant vaginitis severe inflammation to an asymptomatic carrier (1). The other complications are cervical cancer (2), pelvic inflammatory disease (3) and infertility (4). Trichomoniasis may increase the risk of HIV transmission by causing local aggregation of lymphocytes and macrophages of HIV-infected (5).

Molecular method like PCR–RFLP (6), random amplification of polymorphic DNA (RAPD) (7) and PCR-single-strand conformational polymorphism PCR-SSCP (8) to study genetic diversity of the parasite were used in some previous studies.

RAPD as a suitable technique that used random primers for genealogical studies of *T. vaginalis* was proposed in some studies (9–11), PCR-RFLP method are also useful method with sensitivity and reliability that including PCR and RFLP and can display minor changes in a gene (6, 7, 12).

Some *T. vaginalis* isolates are infected with double-stranded RNA (dsRNA) virus belong to the family Totiviridae (13, 14). P270 surface expression is related to the presence of TVV (15). The virus may regulate the transcription of P270 in *T. vaginalis* (16)*.* “The presence TVVs viral infection in *T. vaginalis* is also associated with the expression of cysteine proteinases which are virulence factors” (17). Furthermore, the viruses can induce phenotypic variation in *T. vaginalis* that effect on parasite virulence (18).

Infection of *T. vaginalis* isolates has been reported from all over the world (14, 19–21). The presence of Double-stranded RNA viral infection of *T. vaginalis* (TVV1) has been reported from Iran (22).

There are several studies about epidemiologic, diagnostic, and different aspects of *T. vaginalis,* but (6,7) no study invested on prevalence of the *T. vaginalis* actin genotypes in T*. vaginalis* infected (dsRNA) viruses.

In this study, the prevalence of different genotypes of Iranian isolates of *T. vaginalis* infected (dsRNA) viruses and *T. vaginalis* free of virus was evaluated by PCR-RFLP and obtained patterns were then confirmed by sequence analysis and genotype of these Iranian isolates confirmed again.

## Materials and Methods

### Sample collection

Ten isolates including 8 isolates of *T. vaginalis* collected from previous study and evaluated in points of infection to *T. vaginalis* Virus (TVV-1) were used (22). Two more strains of *T. vaginalis* were collected from 200 vaginal samples of women referred to hospitals associated with Iran University of Medical Sciences in Tehran, Iran during Feb 2016 to Jul 2017.

### Ethical issues

This study was approved by the Ethics Committee of Iran University of Medical Sciences code number (IR.IUMS.REC1395. 95-03-131-28221) in accordance with the Helsinki Declaration and guidelines and all human participation has been obtained in accordance with informed consent.

### Cultivation

Wet mount samples were examined by light microscope, next inoculated into a culture tube at 37 °C in Diamond’s trypticase yeast maltose medium (TYM) with 10% heat-inactivated calf serum, 100 U/mL penicillin and 30 μg/mL streptomycin sulfate. The parasites were harvested in late log phase and centrifuged at 2000×g for 20 min. The pellets were frozen at 70 °C until use.

### Trichomonas vaginalis Virus confirmation RNA Extraction, cDNA Synthesis, PCR

*Trichomonas vaginalis* samples were washed with sterile PBS and centrifuged for 10 min at 4 °C at 5000×g. The RNA was extracted using the Pure Viral Nucleic Acid Kit Roche (Roche Diagnostics GmbH, Mannheim, Germany) according to the manufacturer’s instructions and cDNA Synthesis was carried out and then RT-PCR was performed as previously described (22).

### Molecular typing of the actin gene

#### DNA Extraction

The genomic DNA was extracted from the 10 trichomonads was obtained by culture using the QIAamp DNA minikit (Qiagen, Hilden, Germany),

#### PCR-RFLP

The outer primers and inner primers were selected within an actin gene from the *T. vaginalis* genome (GenBank accession number AF237734) (23). The primers were used as follow:

Tv8S (5′-TCTGGAATGGCTGAAGAAGACG-3′) and Tv9R (5′-CAGGGTACATCGTATTGGTC-3′), and the IPs used were Tv10S (5′-CAGACACTCGTTATCG-3′) and Tv11R (5′-CGGTGAACGATGGATG-3′).

The size of the target was 1100 bp, the PCR mixture consisted of, TaKaRa Ex Taq (5 units/μl), 10X Ex Taq Buffer (Mg2+ free), MgCl_2_ (25 mM), dNTPS Mixture (2.5 mM each), Primers including (Tv8S, Tv9R) and (Tv10S, Tv11R) 0.5μM (final conc.) Sterilized distilled water up to 50 μl.

#### Nested-PCR

The nested PCR was performed in two stages. The first stage consisted of 35 cycles. Each cycle consisted of 30 sec of denaturation at 94 °C, 30 sec of annealing at 55 °C, and 30 sec of extension at 72 °C. The last cycle was followed by a 7 min in final extension at 72 °C. The second stage consisted of 35 cycles with the same denaturation and annealing steps. The PCR products were electrophoresed on a 2% agarose gel. An amplified band of 1100bp corresponded to the actin gene of *T. vaginalis*.

#### RFLP

The restriction endonucleases were used in this study including HindII, RsaI and MseI were purchased from (Thermo Scientific Fast Digest enzymes, California, USA).

After observation of the amplified product, 10 μl of PCR product was digested at 37 °C with restriction endonucleases HindII, RsaI and MseI as described in the manufacture products respectively The PCR product digestion was performed at 37 °C for 4 h for endonucleases HindII, RsaI and about MseI the mixture was digested at 56 °C for 4 h. The fragments were electrophoresed on a 3% agarose gel and obtained band was visualized under UV light. Furthermore different patterns digestion according to DNA fragments of three endonuclease enzymes HindII, RsaI and MseI were compared with previous study (6).

#### Sequencing

To confirm the *T. vaginalis* PCR results, a second round of PCR was done after purification of PCR products using the High Pure PCR Product Purification Kit (Roche Diagnostic, Mannheim, Germany) according to the instructions and were used for direct sequencing using the dye termination method and an ABI 3730xl sequence and all of sequences were analyzed and blasted by Genius software.

The consensus sequences of the sequence types (E, G and I), along with the reference sequences obtained from basic local alignment search tool (BLAST) queries were aligned using version 7.0 of the Genius software.

## Results

Among 1700 vaginal samples were collected 10 *T. vaginalis* was found. The age range was between 15–49 yr with mean of 35 and dominant symptom was vaginal discharge.

### RT-PCR

**Fig. 1:**
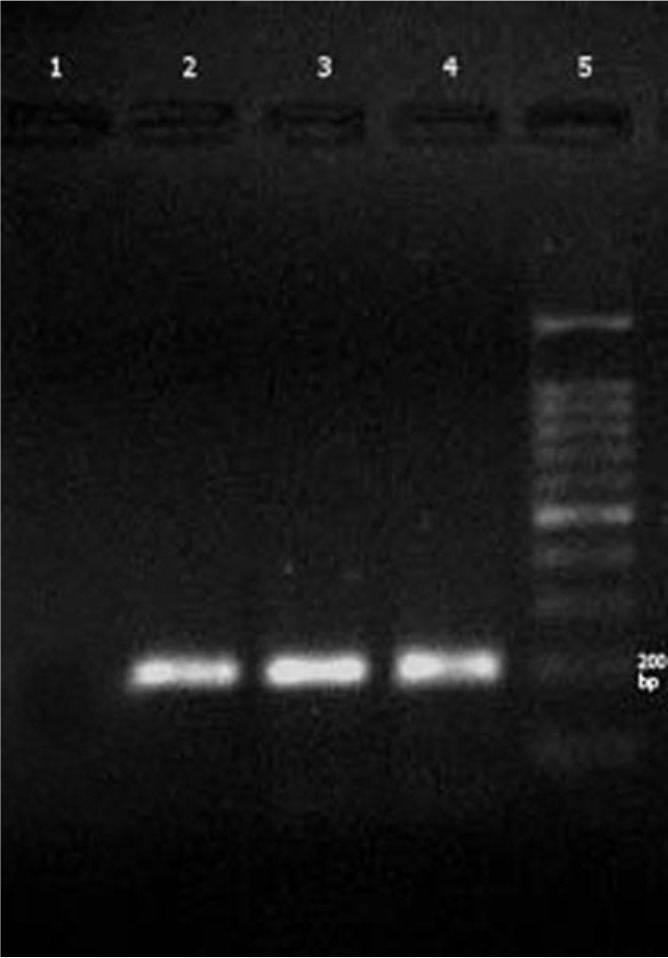
PCR products of TVV1 in Iranian *T. vaginalis* isolates. Lane1: Negative control; 2: Positive control; 3, 4: virus positive *T. vaginalis* samples (204bp); 100 bp DNA ladder marker

Two new isolates of *T. vaginalis* were collected from 200 vaginal samples were negative in points of infection to TVV-1 and overall 10 *T. vaginalis* isolates, only four *T. vaginalis* isolates infected to TVV-1 (Fig. 1) and six isolates were free of TVV-1 infection.

### Nested PCR

**Fig. 2:**
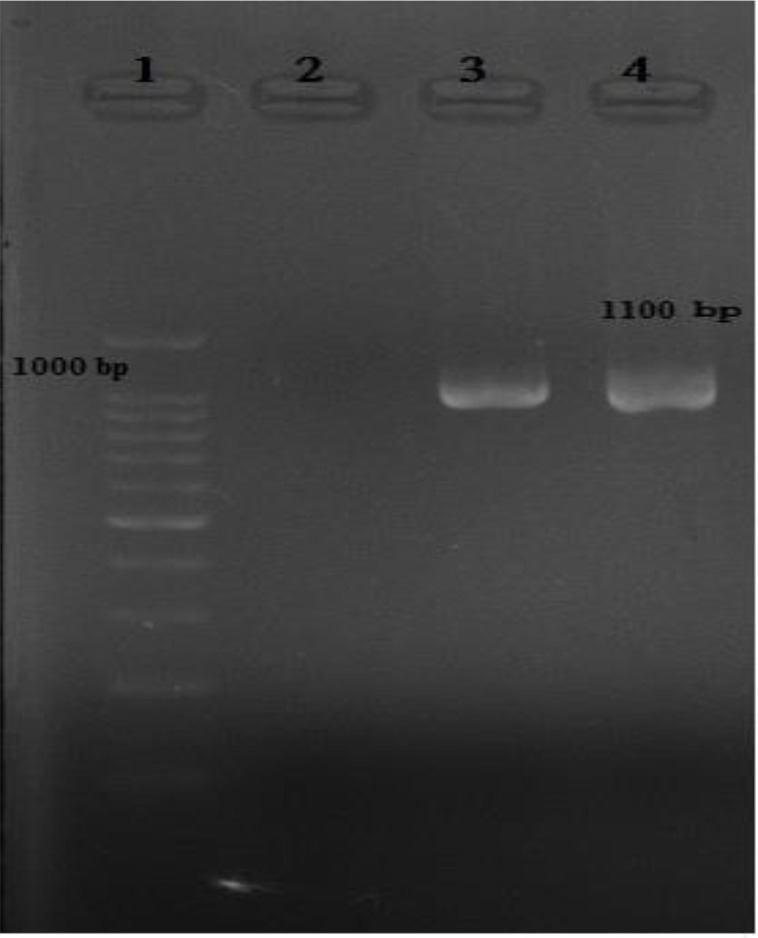
The result of nested –PCR on 2% gel electrophoresis, 1100bp related to actin gene as target was observed.1: marker (100bp), 2: Negative control, 3 and 4: positive samples

All of ten Isolates of *T. vaginalis* were examined by designed nested PCR for actin gene and corresponded 1100 bp band on agarose gel electrophoresis was observed (Fig. 2).

### RFLP Pattern

Overall, different patterns digestion according to DNA fragments of three endonuclease enzymes HindII, MseI and RsaIwere defined. HindII had two patterns, MseI three patterns and RsaI had four patterns

Genotype E was found as presenting pattern 1 after HindII digestion (827,213,60bp) and pattern 2 after MseI (581,315,204bp) and RsaI (568,236,106,103,87bp) digestion. Genotype G was defined as representing pattern 2 after HindII (426,401,213,60bp) and pattern 1 after RsaI (568,236,190,106 bp) and MseI (581,519bp) digestion. Genotype I was defined as showing pattern 2 after HindII (426, 401, 213, 60 bp) and pattern 3 after RsaI (452,236,190,116,106 bp) and 1 after MseI (581,519 bp) digestion.

In this study by combination of fragments pattern of three enzymes of MseI, HindII, and RsaI, three genotypes were found six genotypes E, two genotypes G and two genotypes I. The most dominant genotypes were genotype E.

**Fig. 3:**
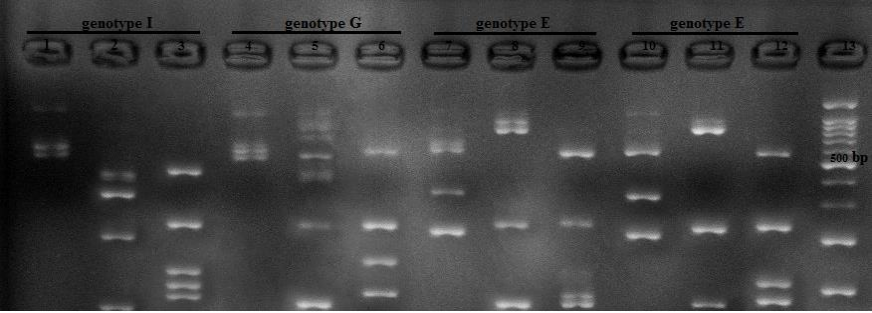
Result of RFLP and separation of the DNA fragments by gel electrophoresis the *T. vaginalis* actin genotypes I, G and E pattern of three enzymes of MseI, HindII, and RsaI respectively

Result of RFLP and separation of the DNA fragments by gel electrophoresis of the *T. vaginalis* actin genotypes I, G and E is shown in Fig. 3.

Among four TVV infected isolates two genotype E, one genotype G and one genotype I were found, however among six uninfected *T. vaginalis* isolates to TVV-1, all of three genotypes were also found.

Result of sequencing all of ten isolates confirmed *T. vaginalis* isolates as below: the sequence corresponded to genotype E had identity to ATCC 50141 (GenBank accession number EU076580), and genotype G had similarity with strains ATCC 30001 (GenBank accession numberEU076578) and genotype I had similarity with isolate N78EU076585.1.

## Discussion

*T. vaginalis* is a sexually transmitted infection all over the world. There are several studies about different aspects of parasite like epidemiology, diagnosis, clinical symptoms, metronidazole resistant, genotype in different regions (6).

The DNA-based techniques are useful method to study epidemiology, genetic diversity of parasite in specific region and population (7).

The several molecular methods have been reported to the study the genetic diversity of parasites (7). These methods like PCR-size polymorphism, PCR-RFLP, RAPD and PCR–hybridization (7).

PCR-RFLP and PCR-RAPD are most useful techniques that have high sensitivity and reliability (7). The PCR-RFLP technique is good performance in strain typing of different organisms like parasites and different bacteria (24).

*T. vaginalis* isolates are infected with dsRNA viruses belong to the family Totiviridae, TVV1 is the most prevalent viral subtype (25, 26). TVV infection is related with the transcriptional upregulation of P270 protein, a phenotypically variable surface immunogen in *T. vaginalis* (16). dsRNA viruses is also associated with the expression of cysteine proteinases known virulence factors (27). Thus, dsRNA viruses can induce various phenotypic changes that impact *T. vaginalis* virulence (18).

The present article describes the application of PCR-RFLP to evaluate Prevalence of the *T. vaginalis* actin genotypes in *T. vaginalis* infected (dsRNA) viruses.

The actin gene was designated as the target in this study. Actin is a protein take part in the filaments formation, it constitutes an important ingredient of the cytoskeleton and plays important role in muscular contraction, motility and *T. vaginalis* changes from the flagellate to pseudopods and may involve in the pathogenesis of parasite (28).

In this study by combination of fragments pattern of three enzymes of HindII, RsaI, and MseI, three genotypes were found as fellow: six genotypes E, two genotypes G and two genotypes I. The most dominant genotypes were genotype E.

The genotypes of E and G have been reported as the most prevalent genotypes in some previous studies (6, 12). In a study the *T. vaginalis* RFLP types were determined for 61 isolates from Kinshasa and 90 isolates from Zambia. Eight different types were identified among the *T. vaginalis* isolates as follow: Genotype E (55.7%, 5.5%), Genotype G (23.0%, 46.7%), H (9.8%, 16.7%) and I (1.6%, 6.7%) in Kinshasa and Zambia respectively. The most common type was type E (55.7% of isolates), in Kinshasa, whereas the most common type was type G (46.7% of isolates) in Zambia (6).

A study was conducted on 45 *T. vaginalis* isolates, to evaluate the genotypes of this parasite using PCR-RFLP based on actin genes in Karaj City, Iran. The prevalence of genotypes were as follow genotype G: 23 (51.1%), genotype E: 11 (24.4%), genotype H: 6(13.3%) and genotype I: 3 (6.6%) (12).

Frequency of *T. vaginalis* among 862 women in Hamadan was reported 16 (1.9%) and three genotypes have been reported including genotype A: 9(56%), I: 6 (38%) and E: 1 (6%) (29).

In a study, 150 vaginal swab and urine specimens were collected and were evaluated using PCR-RFLP on the actin gene. Twenty-four *T. vaginalis* isolates were positive 6 genotypes (H, E, G, I, M, N) were identified. Genotypes H and I were more prevalent (50% and 37.5%) in Kerman and Shiraz, respectively (30).

Among four TVV infected isolates two genotype E, one genotype G and one genotype I were found, however among six uninfected *T. vaginalis* isolates to TVV-1, all of three genotypes were also found.

The result of blasting between the nucleotide sequences which obtained in this study and reference strain were deposited in Gen bank shown, *T. vaginalis* actin genotypes of the strains were determined as genotype E had identity with ATCC 50141 that confirmed as genotype E and isolates were identified as genotype G had identity with strains ATCC 30001 that confirmed as genotype G and genotype I had similarity with EU076585.1 that was genotype I (6).

In a study, polymorphism of *T. vaginalis* was evaluated by PCR-RAPD and position of *T. vaginalis* isolates in phylogenic tree and genetic diversity between different isolates has been studied. The result showed distribution of TVV in tree was random and not in a same cluster group this matter indicates that TVV1 can probably infect virus free strains (9).

Another study was performed on 20 isolates of *T. vaginalis* by PCR-RAPD method. Genealogical tree was constructed. There was no relationship between the presence and absence of the virus in the parasite and genealogic situation of the parasite so the study proposed presence of TVV had no reflection on pattern or clustering of *T. vaginalis* (31).

In confirmation of the mentioned contents, some studies supposed that TVV is associated only with fresh isolates and is lost after prolonged in vitro cultivation. The *T. vaginalis* is acted as harbor and can take or lose the virus several times in its life (32).

Against this matter, Snipes et al evaluated 55 isolates of *T. vaginalis*, which infected to dsRNA virus (TVV) using phylogenetic analysis. The result demonstrated at least two distinct lineages exit that relates to the presence of TVV. Strains harboring the virus are more closely associated with each other than to other *T. vaginalis* population (11).

## Conclusion

Three genotypes E, G and I in *T. vaginalis*, which infected with dsRNA isolates were found, however these three genotypes in *T. vaginalis* without virus were observed.

## References

[1] Petrin D Delgaty K Bhatt R Garber G Clinical and microbiological aspects of *Trichomonas vaginalis*. Clin Microbiol Rev. 1998;11(2):300–17. 9564565 10.1128/cmr.11.2.300 PMC106834

[2] Kharsany AB Hoosen AA Moodley J The association between sexually transmitted pathogens and cervical intra-epithelial neoplasia in a developing community. Genitourin Med. 1993;69(5):357–60. 8244352 10.1136/sti.69.5.357 PMC1195117

[3] Heine P McGREGOR JA *Trichomonas vaginalis*: a reemerging pathogen. Clin Obstet Gynecol. 1993;36(1):137–44. 8435938 10.1097/00003081-199303000-00019

[4] Grodstein F Goldman MB Cramer DW Relation of tubal infertility to history of sexually transmitted diseases. Am J Epidemiol. 1993;137(5):577–84. 8465809 10.1093/oxfordjournals.aje.a116711

[5] Laga M Nzila N Goeman J The interrelationship of sexually transmitted diseases and HIV infection: implications for the control of both epidemics in Africa. AIDS. 1991;5:S55–63. 1669925

[6] Crucitti T Abdellati S Van Dyck E Buvé A Molecular typing of the actin gene of *Trichomonas vaginalis* isolates by PCR–restriction fragment length polymorphism. Clin Microbiol Infect. 2008;14(9):844–52. 18844685 10.1111/j.1469-0691.2008.02034.x

[7] Singh B Molecular methods for diagnosis and epidemiological studies of parasitic infections. Int J Parasitol. 1997;27(10):1135–45. 9394184 10.1016/s0020-7519(97)00111-2

[8] Matini M Rezaie S Mohebali M Genetic identification of *Trichomonas vaginalis* by using the actin gene and molecular based methods. Iran J Parasitol. 2014;9(3):329–35. 25678916 PMC4316563

[9] VAN̂ÁĈOVÁ Ŝ Tachezy J KULDA J FLEGR J Characterization of trichomonad species and strains by PCR fingerprinting. J Eukaryot Microbiol.. 1997;44(6):545–52. 9435127 10.1111/j.1550-7408.1997.tb05960.x

[10] Fraga J Rodriguez J Fuentes O Optimization of random amplified polymorphic DNA techniques for use in genetic studies of Cuban Triatominae. Rev Inst Med Trop Sao Paulo. 2005;47(5):295–300. 16302114 10.1590/s0036-46652005000500010

[11] Snipes LJ Gamard PM Narcisi EM Molecular epidemiology of metronidazole resistance in a population of *Trichomonas vaginalis* clinical isolates. J Clin Microbiol. 2000;38(8):3004–9. 10921968 10.1128/jcm.38.8.3004-3009.2000 PMC87171

[12] Momeni Z Sadraei J Kazemi B Dalimi A Molecular typing of the actin gene of *Trichomonas vaginalis* isolates by PCR-RFLP in iran. Exp Parasitol. 2015;159:259–63. 26542260 10.1016/j.exppara.2015.10.011

[13] Alderete J Wendel K Rompalo A *Trichomonas vaginalis*: evaluating capsid proteins of dsRNA viruses and the dsRNA virus within patients attending a sexually transmitted disease clinic. Exp Parasitol. 2003;103(1–2):44–50. 12810045 10.1016/s0014-4894(03)00068-7

[14] Fraga J Rojas L Sariego I Fernández-Calienes A Double-stranded RNA viral infection in Cuban *Trichomonas vaginalis* isolates. Braz J Infect Dis. 2005;9(6):521–4. 16410949 10.1590/s1413-86702005000600012

[15] Khoshnan A Alderete JF *Trichomonas vaginalis* with a double-stranded RNA virus has upregulated levels of phenotypically variable immunogen mRNA. J Virol. 1994;68(6):4035–8. 8189538 10.1128/jvi.68.6.4035-4038.1994 PMC236912

[16] Bessarab IN Liu HW Ip CF Tai JH The complete cDNA sequence of a type II *Trichomonas vaginalis* virus. Virology. 2000;267(2):350–9. 10662630 10.1006/viro.1999.0129

[17] Provenzano D Khoshnan A Alderete JF Involvement of dsRNA virus in the protein compositionand growth kinetics of host *Trichomonas vaginalis*. Arch Virol. 1997;142(5):939–52. 9191859 10.1007/s007050050130

[18] Bessarab IN Nakajima R Liu HW Tai JH Identification and characterization of a type III *Trichomonas vaginalis* virus in the protozoan pathogen *Trichomonas vaginalis*. Arch Virol. 2011;156(2):285–94. 21110050 10.1007/s00705-010-0858-y

[19] Weber B Mapeka T Maahlo M Hoosen A Double stranded RNA virus in South African *Trichomonas vaginalis* isolates. J Clin Pathol. 2003;56(7):542–543. 12835302 10.1136/jcp.56.7.542 PMC1770003

[20] El-Gayar EK Mokhtar AB Hassan WA Molecular characterization of double-stranded RNA virus in *Trichomonas vaginalis* Egyptian isolates and its association with pathogenicity. Parasitol Res. 2016; 115(10):4027–36. 27316695 10.1007/s00436-016-5174-3

[21] Wendel KA Rompalo AM Erbelding EJ Double-stranded RNA viral infection of *Trichomonas vaginalis* infecting patients attending a sexually transmitted diseases clinic. J Infect Dis. 2002;186(4):558–61. 12195385 10.1086/341832

[22] Khanaliha K Masoumi-Asl H Bokharaei-Salim F Double-stranded RNA viral infection of *Trichomonas vaginalis* (TVV1) in Iranian isolates. Microb Pathog. 2017; 109:56–60. 28478201 10.1016/j.micpath.2017.04.032

[23] Espinosa N Hernández R Lopez-Griego L Arroyo R Lopez-Villasenor I Differences between coding and non-coding regions in the *Trichomonas vaginalis* genome: an actin gene as a locus model1. Acta Trop. 2001;78(2):147–54. 11230824 10.1016/s0001-706x(00)00180-7

[24] Pillay A Liu H Chen CY Holloway B Sturm WA Steiner B Molecular Subtyping of *Treponema pallidum* Subspecies pallidum. Sex Transm Dis. 1998;25(8):408–14. 9773432 10.1097/00007435-199809000-00004

[25] Goodman RP Ghabrial SA Fichorova RN Nibert ML Trichomonas virus: a new genus of protozoan viruses in the family Totiviridae. Arch Virol. 2011;156(1):171–9. 20976609 10.1007/s00705-010-0832-8 PMC3659425

[26] Fraga J Rojas L Sariego I Fernández-Calienes A Genetic characterization of three Cuban *Trichomonas vaginalis* virus. Phylogeny of Totiviridae family. Infect Genet Evol. 2012;12(1):113–20. 22075038 10.1016/j.meegid.2011.10.020

[27] Sommer U Costello CE Hayes GR Identification of *Trichomonas vaginalis* cysteine proteases that induce apoptosis in human vaginal epithelial cells. J Biol Chem. 2005;280(25):23853–60. 15843376 10.1074/jbc.M501752200

[28] Bricheux G Brugerolle G Molecular cloning of actin genes in *Trichomonas vaginalis* and phylogeny inferred from actin sequences. FEMS Microbiol Lett. 1997;153(1):205–13. 9252588 10.1111/j.1574-6968.1997.tb10483.x

[29] Matini M Rezaei H Fallah M Genotyping, Drug Susceptibility and Prevalence Survey of *Trichomonas vaginalis* among Women Attending Gynecology Clinics in Hamadan, Western Iran, in 2014–2015. Iran J Parasitol. 2017;12(1):29–37. 28761458 PMC5522696

[30] Oliaee Rt Babaei Z Hatam Gr Considerable Genetic Diversity of *Trichomonas vaginalis* Clinical Isolates in a Targeted Population in South of Iran. Iran J Parasitol. 2017;12(2):251–9. 28761486 PMC5527036

[31] Hampl V Vaňáčová Š Kulda J Flegr J Concordance between genetic relatedness and phenotypic similarities of *Trichomonas vaginalis* strains. BMC Evol Biol. 2001;1:11. 11734059 10.1186/1471-2148-1-11 PMC60492

[32] Wang A Wang CC Alderete JF *Trichomonas vaginalis* phenotypic variation occurs only among trichomonads infected with the double-stranded RNA virus. J Exp Med. 1987;166(1):142–50. 3298522 10.1084/jem.166.1.142 PMC2188647

